# Glycogenic Hepatopathy: A Rare Hepatic Complication of Poorly Controlled Type 1 DM

**DOI:** 10.1155/2020/1294074

**Published:** 2020-04-13

**Authors:** Leila A. Alenazy, Muhammad Javed, Hussien Elsiesy, Emad Raddaoui, Waleed K. Al-Hamoudi

**Affiliations:** ^1^Department of Gastroenterology and Hepatology Unit (59), Department of Medicine, King Saud University, Riyadh 11461, Saudi Arabia; ^2^Department of Liver Transplantation, King Faisal Specialist Hospital, Alfaisal University, Riyadh, Saudi Arabia; ^3^Department of Pathology, Alfaisal University, Riyadh, Saudi Arabia

## Abstract

Glycogen hepatopathy (GH) is a rare complication of type 1 diabetes mellitus that leads to an abnormal accumulation of glycogen in the hepatocytes. The exact mechanism of GH remains unknown, but fluctuations in blood glucose and insulin levels play important roles in promoting glycogen accumulation. We report a case of a 16-year-old female diagnosed with poorly controlled type 1 diabetes mellitus with hepatomegaly and elevated liver enzymes. The patient experienced multiple admissions for diabetic ketoacidosis, and she also had celiac disease diagnosed 2 years previously based on serology and a duodenal biopsy. The laboratory analyses results were compatible with acute hepatitis, and the celiac serology was positive. Other investigations ruled out viral hepatitis and autoimmune and metabolic liver diseases. Ultrasound and computerized tomography (CT) scans of the abdomen revealed liver enlargement with diffuse fatty infiltration. A liver biopsy revealed the presence of abundant glycogen in the cytoplasm of the hepatocytes. PAS staining was strongly positive, which confirmed the diagnosis of GH. There were no features of autoimmune hepatitis or significant fibrosis. Duodenal biopsy results were consistent with celiac disease. Despite our efforts, which are supported by a multidisciplinary team approach that included a hepatologist, a diabetic educator, a dietitian, and an endocrinologist, we have encountered difficulties in controlling the patient's diabetes, and she persistently maintains symptomatic hepatomegaly and abnormal liver biochemistry. Given the patient's age, we assumed that these abnormalities were related to patient noncompliance. In conclusion, GH remains an under-recognized complication of type 1 DM that is potentially reversible with adequate glycemic control. The awareness of GH should prevent diagnostic delay and its implications for management and the outcome.

## 1. Introduction

Glycogen hepatopathy is characterized by glycogen deposition in hepatocytes due to both glycogen synthesis and the inhibition of glycogenolysis. This condition has been described in type 1 diabetes mellitus and occurs as a result of an imbalance in the production and degradation of glycogen following insulin introduction. This imbalance leads to the abnormal accumulation of glycogen in hepatocytes and results in hepatomegaly and the leakage of transaminases out of liver cells [1–3]. Although the mechanism of the development of GH is still unrecognized, it is clear that fluctuations in blood glucose and insulin levels play an important role in promoting glycogen accumulation [4].

We report a case of a 16-year-old female diagnosed with poorly controlled type 1 diabetes mellitus (T1DM) on insulin therapy. She was referred to an adult hepatology outpatient clinic due to hepatomegaly and elevated liver enzymes that were discovered incidentally during a routine workup a year previously.

## 2. Case Report

A 16-year-old female was referred to an adult hepatology outpatient clinic due to elevated liver enzymes that were discovered incidentally during a routine workup one year previously. She had T1DM diagnosed 4 years previously and was receiving insulin therapy with an average requirement of 1.2 units/kg/day. However, her diabetes was poorly controlled (hemoglobin A_1C_ 11.5%), and she had multiple admissions for diabetic ketoacidosis.

She was frequently nauseated and frequently complained of abdominal distension associated with mild to moderate colicky intermittent epigastric pain. There was no history of liver disease, blood transfusion, herbal ingestion, or cholelithiasis. She was not an alcohol or drug consumer and was not taking any medication other than insulin. Additionally, she did not exhibit any symptom or signs compatible with acute or chronic hepatitis. The patient had celiac disease diagnosed 2 years previously based on serology and a duodenal biopsy. She was on a strict gluten-free diet. Her elder brother had T1DM, and her younger brother had eczema. There was no family history of liver diseases or other autoimmune disorders. Her menarche occurred at 12 years of age, and her periods were irregular.

Physical examination revealed a body mass index of 22.7 kg/m^2^. She had a nontender distended and tense abdomen with hepatomegaly. She had no ascites or stigmata of chronic liver disease. Her pubertal development was normal. The physical examination was otherwise unremarkable.

**Figure 1:**
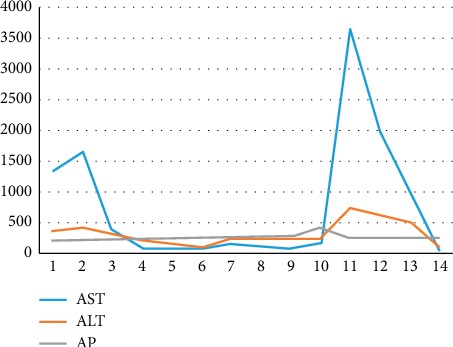
Liver enzymes pattern.

During her clinical follow-ups, she had several severe flares of serum transaminases that returned to normal within days without any specific treatment. Her laboratory analyses were compatible with acute hepatitis (Figure 1) with concomitant increases in gamma-glutamyl transferase (164 U/L, normal <55) and alkaline phosphatase (286 U/L, normal <180). Her liver function panels were all normal and included albumin (39 g/L), INR (0.81), and total bilirubin (4 *µ*mol/L). Her lipid profile was abnormal with elevated triglycerides (5.26 mmol/L) and a cholesterol level of 7.2 mmol/L.

A complete blood cell count, renal profile, and ferritin level were normal. The serologies for viral hepatitis A, B, C, E, HIV, EBV, and cytomegalovirus were negative. A complete workup for autoimmune and metabolic liver disease was unremarkable and included tests for serum ceruloplasmin, copper, alpha 1-antitrypsin, alpha-fetoprotein, and immunoglobulins. Additionally, anti-nuclear antibody (ANA), anti-nuclear smooth muscle antibody (ASMA), anti-mitochondrial antibody (AMA), and liver-kidney microsomal antibody (LKM1) were all negative. The celiac serology was positive. Her thyroid function, FSH, LSH, and prolactin levels were normal. Her vitamin D level was low (58.29 nmol/l normal >145), and her vitamin B12 and folate levels were normal.

**Figure 2:**
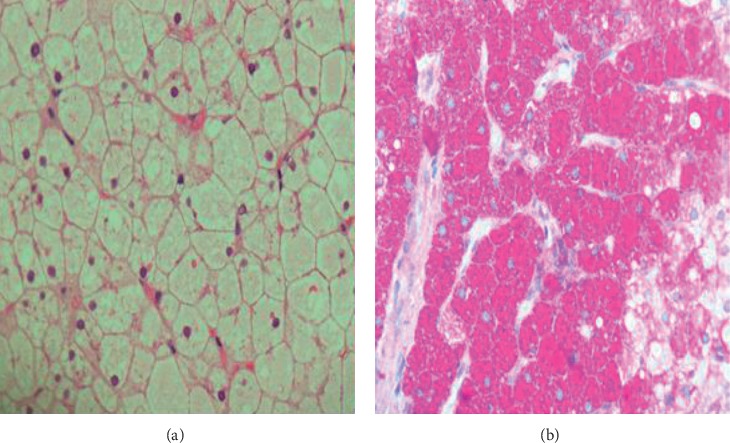
(a) Liver biopsy showing presence of abundant glycogen in the cytoplasm of hepatocytes and (b) a strongly positive PAS stain.

The ultrasound and CT scans of the abdomen revealed liver enlargement (23 cm span) with a bright, coarse echotexture. She exhibited no splenomegaly or ascites. A liver biopsy revealed the presence of abundant glycogen in the cytoplasm of the hepatocytes. The PAS staining was strongly positive. Perls staining revealed no iron deposition. There were no features of autoimmune hepatitis or significant fibrosis (Figure 2). A duodenal biopsy revealed total villous atrophy with an increase in intraepithelial lymphocytes and crypt hyperplasia consistent with celiac disease.

Despite our efforts, which were supported by a multidisciplinary team approach that included a hepatologist, diabetic educator, dietitian, and endocrinologist, we have encountered difficulties in controlling the patient's diabetes, and she is persistently symptomatic with hepatomegaly and abnormal liver biochemistry. Given the patient's age, we initially assumed that these abnormalities were related to patient noncompliance.

## 3. Discussion

From a historical point of view, in 1930, Pierre Mauriac described Mauriac Syndrome (MS) as a rare complication of poorly controlled type I DM in children. MS is characterized by overloading of the hepatocytes with glycogen, hepatomegaly, and abnormal liver enzymes. MS is associated with growth failure, delayed puberty, and cushingoid features. These complications were first described shortly after the introduction of insulin in 1922 [1].

Hepatic glycogen deposition was described as a feature of MS, but more recently it has been described in the absence of other MS features. Even more recently, this condition has been known as glycogenic hepatopathy (GH) and has been described in children, adolescents, and young adult with poorly controlled type I DM. MS has recently been reported in patients with type II DM who are receiving insulin therapy [1–3].

The disease results from glycogen synthesis and the inhibition of glycogenolysis. In the state of hyperglycemia, glucose enters hepatocytes independent of insulin and is subsequently converted into glucose-6-phosphate (G-6-P) by glucokinase. This high level of G-6-P can allosterically stimulate glycogen synthesis via the activation of glycogen synthase. When insulin is administered, it has a covalent modification effect on both glycogen synthesis and glycogenolysis that involves the promotion of the dephosphorylation of both the glycogen phosphatase and glycogen synthase enzymes. This effect leads to an imbalance in the production and degradation of glycogen, and the hepatocytes subsequently abnormally accumulate glycogen. Swelling of the hepatocytes leads to the leakage of liver transaminases and hepatomegaly. This condition can occur as early as two to four weeks after the initiation of insulin therapy or as a late complication of poorly controlled insulin-dependent diabetes mellitus. However, not all diabetic patients who exhibit poor glycemic control develop GH. It has been suggested that a regulatory protein is responsible for the marked glycogen accumulation [5]; however, recent studies have failed to demonstrate such an effect [4, 6–8].

Although the mechanism of the development of GH is still unrecognized, it is clear that fluctuations in blood glucose and insulin levels play an important role in the promotion of glycogen accumulation. This process can be controlled by either stabilizing the persistent hyperglycemia with infrequent large doses of insulin, such as those given during ketoacidosis, or alternately by the administration of glucose to patients who are receiving excessive insulin to treat the hypoglycemic episodes [4, 8].

Several studies have demonstrated that intensive glycemic control or the normalization of glucose metabolism in post-pancreatic transplantation leads to the normalization of liver enzymes and resolves hepatomegaly. Additionally, the complete resolution of histopathological changes can occur within a relatively short period, which results in a favorable prognosis [7–9].

Hepatomegaly and elevated serum transaminase in diabetic patients are commonly associated with nonalcoholic fatty liver disease (NAFLD). Clinically, it is important to distinguish GH from NAFLD. The characteristics of both diseases are summarized in Table 1 [2–4, 9, 10].

In our case, prior to obtaining the liver biopsy, we thought of celiac hepatopathy as a possible explanation for her unexplained elevated liver enzymes given her positive serology and abnormal duodenal biopsy. Our case is one of the two reported cases of GH in association with celiac disease. Celiac hepatopathy clinically, radiologically, and histologically varies depending on the degree of liver injury. The characteristics of celiac hepatopathy are summarized in Table 2 [11, 12].

## 4. Conclusion

GH remains an under-recognized complication of type 1DM and is characterized by severe transaminase flares and hepatomegaly and is reversible with adequate glycemic control. Clinicians should monitor the clinical response to optimize the glycemic control. Furthermore, the awareness of GH should prevent diagnostic delay and its implications for management and outcome.

## Figures and Tables

**Table 1:** Comparison of nonalcoholic fatty liver disease and glycogenic hepatopathy.

	Nonalcoholic fatty liver disease (NAFLD)	Glycogenic hepatopathy (GH)
*Clinically*	*Symptoms*: asymptomatic	*Symptoms*: mild epigastric pain, nausea, and vomiting.
	*Signs*: nontender hepatomegaly	*Signs*: hepatomegaly ± tenderness.
	*Liver enzymes*: mild to moderate (less than 5 times the upper normal limit), persistent elevated liver enzymes	*Liver enzymes*: severe flares of transaminases reaching up to 2000–4000 U/L
	*Liver function panel*: depends on the degree of liver injury	*Liver function panel*: normal

*Radiological*	*Abdominal CT scan*: reduced hepatic CT attenuation due to fat deposition in the liver (low density)	*Abdominal CT scan*: increased hepatic CT attenuation due to glycogen deposition in to liver (high density)
	*Gradient-dual-echo MRI* can be considered a powerfully noninvasive tool for identification	*Gradient-dual-echo MRI* can be considered a powerfully noninvasive tool for identification
	(i) High intensity on subtraction	(i) Low intensity on subtraction

*Histologically*	*Histological finding*: (for a definitive diagnosis) Ranges from (i) steatosis alone (ii) to nonalcoholic steatohepatitis (NASH) with varying risks of progression to cirrhosis	*Histological finding*: (for definitive diagnosis) (i) swollen hepatocytes and pale cytoplasm (ii) abundant cytoplasmic glycogen deposits are demonstrated by periodic acid-Schiff (PAS) staining and glycogen removal is demonstrated by diastase digestion (iii) no evidence of necrosis, inflammation, steatosis, or fibrosis

*Pathogenesis*	(i) Common in T2DM and T1DM, regardless of insulin therapy	(i) Common in T1DM and rare in T2DM with insulin therapy

*Treatment Prognosis*	(i) Can progress to fibrosis and cirrhosis	(i) No progression to fibrosis or cirrhosis
	(ii) Optimize treatment of risk factors and lifestyle modification	(ii) Reversible with adequate glycemic control

**Table 2:** Comparison of celiac hepatopathy and glycogenic hepatopathy.

	Celiac hepatopathy (CH) [10, 11]	Glycogenic hepatopathy (GH)
*Clinically*	*Symptoms*: asymptomatic, mild symptoms commonly malaise and fatigue, or symptoms of severe liver failure	*Symptoms*: mild epigastric pain, nausea, and vomiting
	*Sign*s: range from normal physical examination to liver failure	*Signs*: hepatomegaly ± tenderness
	*Liver enzymes*: mild to moderate (less than 5 times the upper normal limit)	*Liver enzymes*: severe flares of transaminases reaching up to 2000–4000 U/L
	*Liver function panel*: depends on the degree of liver injury	*Liver function panel*: normal

*Radiological*	*Abdominal ultrasound*: normal to coarse echo texture associated with findings that suggest the activity or suspension of unrecognized CD as	*Abdominal ultrasound*
		Hepatomegaly measured around (17–23 cm) bright coarse echotexture
	(i) dilated small bowel loops	*Abdominal CT scan*: hepatomegaly and high-density and marked attenuation
	(ii) enlarged mesenteric lymph nodes	
	(iii) increased peristalsis	
	(iv) abnormal jejunum folds	
	(v) enlarged mesenteric lymph nodes	

*Histologically*	*Histological findings*:	*Histological findings*:
	(i) Nonspecific, most commonly periportal inflammation, mononuclear infiltration of the parenchyma, bile duct obstruction, hyperplasia of the Kupffer cells	(i) Swollen hepatocytes and pale cytoplasm
	(ii) Steatosis	(ii) Abundant cytoplasmic glycogen deposits are demonstrated by periodic acid-Schiff (PAS) staining, and glycogen removal is demonstrated by diastase digestion
	(iii) Less common, advanced lesions with fibrosis and liver cirrhosis. Fibrosis (all stages), and cirrhosis	(iii) No evidence of necrosis, inflammation, steatosis, or fibrosis

*Treatment prognosis*	Strict gluten-free diet (GFD)	Glycemic control
	(i) Can progress to fibrosis and cirrhosis	(i) No progression to fibrosis or cirrhosis
	(ii) Reversible with strict GFD	(ii) Reversible with adequate glycemic control

*Duration needed for complete resolution clinically, radiological, and histologically*	(i) Six months to one year	(i) Depends on the achievement of adequate glycemic control but can be as early as four to five weeks
	(ii) Reversibility is considered pathognomonic to celiac hepatitis	
